# Percutaneous Closure of Patent Foramen Ovale after Anterior Spinal Cord Infarction

**DOI:** 10.1155/2022/2171350

**Published:** 2022-04-20

**Authors:** João Ferreira Reis, Petra Loureiro, Rita Lopes Silva, José Diogo Martins

**Affiliations:** ^1^Department of Cardiology, Hospital de Santa Marta, Centro Hospitalar Universitário de Lisboa Central, Lisbon, Portugal; ^2^Department of Pediatric Cardiology, Hospital de Santa Marta, Centro Hospitalar Universitário de Lisboa Central, Lisbon, Portugal; ^3^Pediatric Neurology Unit, Pediatric Department, Hospital de Dona Estefânia, Centro Hospitalar Universitário de Lisboa Central, Lisbon, Portugal

## Abstract

In patients with a patent foramen *ovale* (PFO) who have had a cryptogenic ischemic stroke, percutaneous closure reduces its recurrence risk. However, its role in spinal cord infarction (SCI) is less well-established. A few case reports describe the putative causative role of PFO in SCI. We present a case of a teenager with cryptogenic anterior SCI in the setting of a deep vein thrombosis and a high risk-PFO who underwent successful percutaneous closure.

## 1. Introduction

Spinal cord infarction is an uncommon, but important cause of acute myelopathy; however, a definite cause is not found in a significant proportion of cases (60–75% of the cases) [1, 2]. The presence of a patent foramen *ovale* is a well-established risk factor for cryptogenic brain infarction due to paradoxical embolism; however, the physiopathological link of right-to-left shunting in spinal cord ischemia is less clearly defined.

## 2. Case Report

### 2.1. History of Presentation

We describe the case of a 16-year-old male patient who presents to the emergency department with cervical pain radiating to both arms, followed by bilateral upper limb weakness and numbness, which began after performing a Valsalva maneuver (intense physical exercise—abdominal crunches). The paresthesias rapidly progressed to the abdomen and lower extremities, despite intact lower limb strength, and sphincter dysfunction developed. He denied having suffered a relevant trauma in the previous days.

A comprehensive neurological exam revealed flaccid paralysis of both upper extremities, with muscle power of 2/5 on the Medical Research Council (MRC) scale in 3 upper right arm adduction and right forearm flexion and muscle power 3/5 in upper left arm adduction and left forearm flexion, downward drift of both outstretched arms (Barré test), bilateral bicipital hyperreflexia, and pain and temperature sensation impairment in both upper and lower extremities. Proprioception, lower extremity strength, and cranial nerve examination were normal.

### 2.2. Past Medical History

The patient is overweight (body mass index -32 kg/m^2^) and had a right tibial fracture in the previous year, treated with osteosynthesis. There is familial history of venous thromboembolism and premature atherosclerotic disease.

### 2.3. Differential Diagnosis

The clinical presentation is that of a “man-in-the-barrel syndrome,” which translates an anterior spinal cord syndrome and can have several possible etiologies, including ischemic, inflammatory, traumatic, or neoplastic.

### 2.4. Investigations

Laboratory tests were remarkable for a borderline positive antinuclear antibodies' titer of 1 : 160 (speckled pattern) with positive anti-DFS70 autoantibodies, but the remainder of the autoimmune workup was negative, including antiphospholipid antibodies and high factor VIII levels (182%). The workup was otherwise normal, including hypercoagulability, vasculitis, infectious, and transverse myelitis workups were negative. Cerebrospinal fluid examination was unremarkable.

**Figure 1:**
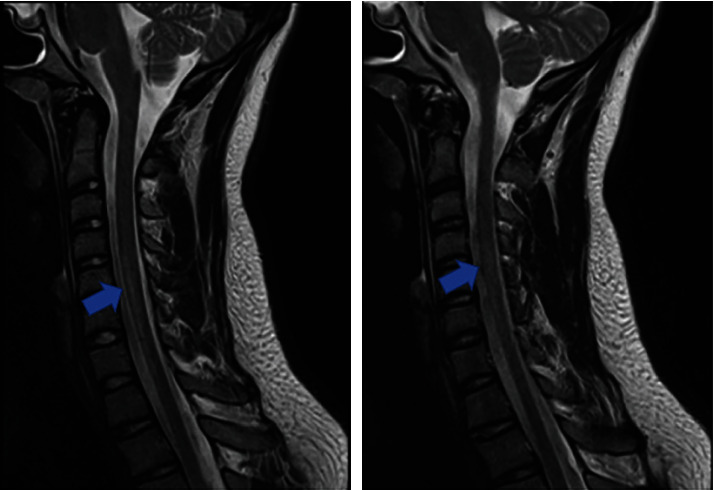
Spine MRI showing an extensive multisegmental T2-weighted hyperintense signal between the median plane of C4 and the transition from C7 to D1 (arrows) with symmetrical anterior medullary involvement and restricted diffusion on DWI, but without spinal cord expansion or signs of spinal cord compression. There was a normal T2 vertebral artery flow void, with no apparent signs of acute vertebral artery dissection or intramural hematoma.

T2-weighted sagittal MRI of the cervical spine disclosed a hyperintense lesion extending from C4 to D1 involving the anterior horns of the spinal cord, without signs of spinal cord compression and no apparent signs of acute vertebral artery dissection or intramural hematoma (Figure 1). Brain MRI was unremarkable.

Chest and CT angiography of supra-aortic arteries failed to identify an etiology and excluded acute aortic syndrome or supra-aortic vessel dissection. Digital subtraction spinal and supra-aortic artery angiography excluded abnormalities of the spinal cord vascular system, namely, arteriovenous malformations and vessel dissection, despite a slow filling of the anterior spinal artery. Lower Extremity Venous Doppler Study revealed thrombotic occlusion of the left posterior tibial artery.

Transcranial Doppler showed patency of major cerebral arteries, and saline bolus detected a right-to-left at-rest grade I shunt according to the Spencer grading scale during rest that increased with Valsalva maneuver. In order to evaluate for the presence of an intracardiac shunt, the patient underwent a transesophageal echocardiogram (TEE) that revealed the presence of a PFO with a tunnel length of 18 mm and an additional 2 mm ostium secundum atrial septal defect and an exuberant persisting Eustachian valve.

### 2.5. Management

Upon diagnosis of deep venous thrombosis, anti-Xa assay-guided low molecular weight heparin was started, requiring multiple dose adjustments due to subtherapeutic levels. The patient underwent successful physical rehabilitation.

**Figure 2:**
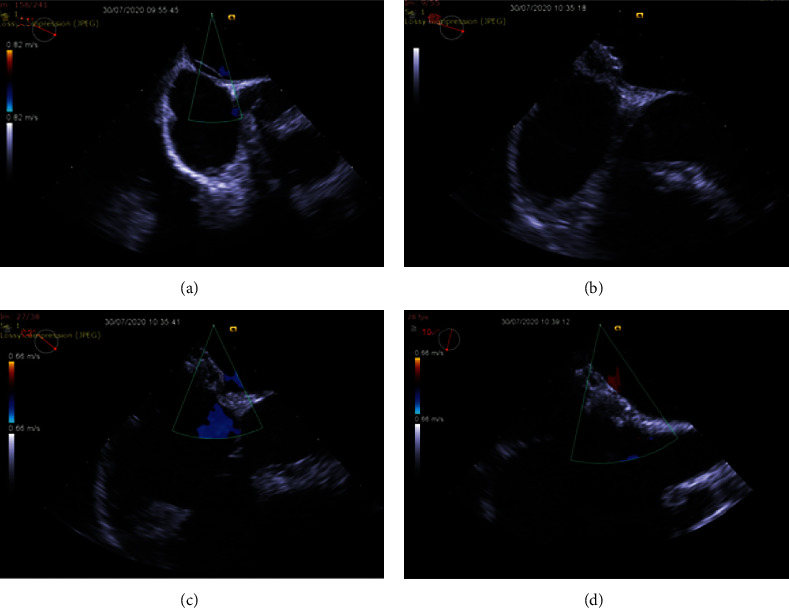
(a) TEE revealing a long-tunneled PFO. (b–d) TEE showing the final position of Cardia Ultrasept PFO 20 mm device, with no residual leak.

**Figure 3:**
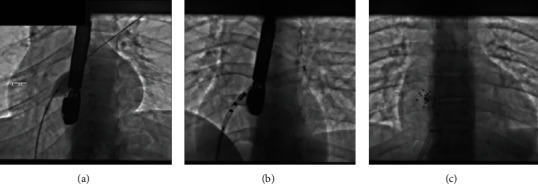
(a–c) Cardia Ultrasept PFO 20 mm device implantation.

The patient was referred to our cardiac center for percutaneous closure (PC). Under general anesthesia and TEE guidance, a Cardia Ultrasept PFO 20 mm device was successfully implanted, with no residual leak (Figures 2 and 3). The procedure was undertaken 8 months after spinal cord infarction, and a lower extremity venous Doppler was repeated prior to PFO closure in order to document resolution of deep venous thrombosis and ensure the safety of the procedure.

Due to a probable underlying thrombophilia, it was decided to maintain anticoagulation.

## 3. Discussion

We report one of the first percutaneous closure of a PFO in a patient with a cryptogenic medullar infarction, involving the youngest patient described in literature.

PFO affects about 25% of the adult population. It has a role in cryptogenic stroke in young adults, by providing a conduit to paradoxical embolism or by serving as a nidus for *in situ* thrombus formation, particularly in long-tunneled PFO with exuberant Eustachian valves. Recent randomized controlled trials (RCTs) [3–6] have demonstrated superiority of PC in comparison with medical therapy alone for prevention of recurrent stroke.

The vast majority of strokes associated with PFO are of cortical pattern, and the major RCTs evaluating the efficacy and safety of its percutaneous closure excluded patients with strokes due to small-vessel occlusive disease. Spinal cord infarction (SCI) due to paradoxical embolism is rare, and a few recent case reports [7–12] have postulated a possible physiopathological link between PFO-mediated paradoxical embolism and SCI.

We debated carefully the decision to close our patient's PFO. Contrary to closure, patients under 18 years old are largely underrepresented or have been excluded from the major RCTs [3–6]; there is tenuous clinical data that support this intervention after cryptogenic SCI; the long-term arrhythmic burden associated with PC including permanent postprocedural atrial fibrillation (AF) is largely unknown. Data on AF after transcatheter PFO closure are sparse; however, according to a recent meta-analysis, PFO closure increased the risk of new-onset AF at 4.6 times compared to medical therapy (4.2% vs. 0.7%; risk ratio 4.55, CI 95% 2.16-9.6, and *p* = 0.0001) [13]. In the REDUCE clinical study, postprocedural AF was mostly early onset, transient, and with no later recurrence and occurred more frequently among patients with higher age and larger devices [14].

Favoring closure, our patient presented with several features highly suggestive of a PFO-related paradoxical embolic event: the presence of lower limb deep venous thrombus; the temporal association between a Valsalva maneuver and symptom onset; the presence of high-risk characteristics associated with the PFO (exuberant Eustachian valve, long tunnel) [15], and, finally, the elevated levels of factor VIII, which are an established independent risk factor for thrombotic events, especially if the levels are above 150% [16].

## 4. Follow-Up

In the short-term follow-up, the patient is doing well, with no additional neurological events.

## 5. Conclusions

Our case describes the first percutaneous PFO closure in SCI in a young patient after a deep vein thrombosis and with a positive family history for venous thromboembolism. Due to the lack of randomized controlled trials on the management of SCI in the presence of PFO, the decision to close the PFO was not straightforward. However, the clinical context suggestive of paradoxical embolism, a probable underlying prothrombotic predisposition, the high-risk features of the PFO, and operator's experience played a role in the decision to close the PFO, despite patient's young age. Our work seeks to raise awareness to the role of right-to-left shunts and paradoxical embolism in cryptogenic ischemic spinal disorders.

## Data Availability

The data used to support the findings of this study are included within the article.

## Additional Points

*Learning Objectives*. (i) To recognize patent foramen *ovale* (PFO) and other right-to-left shunts mediated paradoxical embolism as a cause of spinal cord infarction (SCI). (ii) To be able to identify the ideal candidates for percutaneous PFO closure.
